# Supplementary material for: Higher-order connectomics of human brain function reveals local topological signatures of task decoding, individual identification, and behavior
Source: Nat Commun. 2024 Nov 26;15:10244. doi: 10.1038/s41467-024-54472-y (PMC11599762; doi:10.1038/s41467-024-54472-y)
Supplement: Supplementary file 1 — Supplementary Information [file 41467_2024_54472_MOESM1_ESM.pdf]

# Supplementary Material: Higher-order connectomics of human brain function reveals local topological signatures of task decoding, individual identification, and behavior

Andrea Santoro[1,2], Federico Battiston[3], Maxime Lucas[2,4], Giovanni Petri[5,6,2], Enrico Amico[1,7,8]
[1]Neuro-X Institute, EPFL, Geneva, Switzerland
[2]CENTAI, Corso Re Inghilterra 3, Turin, 10138, Italy
[3]Department of Network and Data Science, Central European University, 1100 Vienna, Austria
[4]Department of Mathematics & Namur Institute for Complex Systems (naXys), Université de Namur, Belgium
[5]NPLab, Network Science Institute, Northeastern University London, London, E1W 1LP, UK
[6]Department of Physics, Northeastern University, Boston, MA, 02115, USA
[7]School of Mathematics, University of Birmingham, Birmingham, B15 2TS, UK
[8]Centre for Human Brain Health, University of Birmingham, Birmingham, B15 2TT, UK

September 19, 2024

## Contents

**S1 Task decoding** 1

**S2 Brain Fingerprinting** 3

**S3 Partial Least Square Correlation (PLSC) and Canonical Correlation Analysis (CCA)** 4

**S4 Comparison with multivariate information-theoretic approaches** 7
- S4.1 Cortical profiles . . . 8
- S4.2 Functional brain fingerprinting . . . 8
- S4.3 Brain-behavior association . . . 8

**S5 ConnICA comparison** 11

## S1 Task decoding

In the main text, we have shown the task decoding ability of the four methods on resting-state and task fMRI data of the HCP dataset. In particular, once the time-time correlation matrices have been computed, we binarize the matrices by considering a common threshold at the $95th$ percentile. While this choice provides the highest task decoding ability in terms of the element-centric similarity (ECS) by all methods, we report in Fig. S1-S2 the same plots reported in the main text but when considering two other thresholds, namely, $90th$ and $97th$ percentiles.

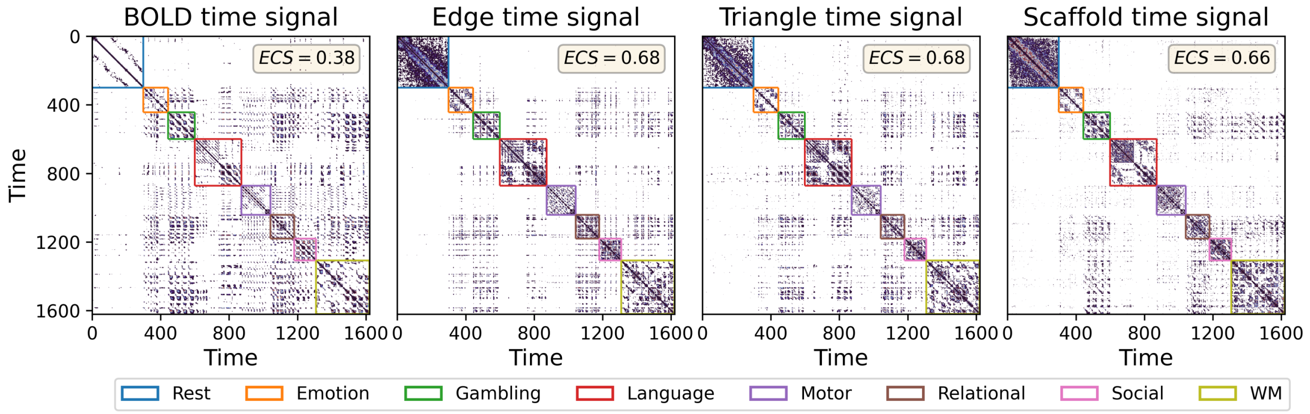


Figure S1: **Local higher-order topological indicators for fMRI task differentiability at** $90th$ **percentile**. As done in the main text, we compute *local* observables by comparing the temporal recurrence plots (i.e. time-time correlation matrices) for the four methods, from the lower-order methods (BOLD and edge signals) to higher-order ones (triangle and scaffold time signals). We set a common threshold at the $90th$ percentile to binarize the data when analyzing an fMRI temporal signal obtained by concatenating resting-state and seven HCP tasks. Colored boxes within the plots denote the ground truth of rest and task segments. The element-centric similarity (ECS) measure provides a quantification of the task decoding ability of the different methods. Here, edges and triangles lead the pack, yet with a lower value than the one reported in the main text. Results are averaged over the 100 subjects, considering the mean between LR and RL phase encoding.

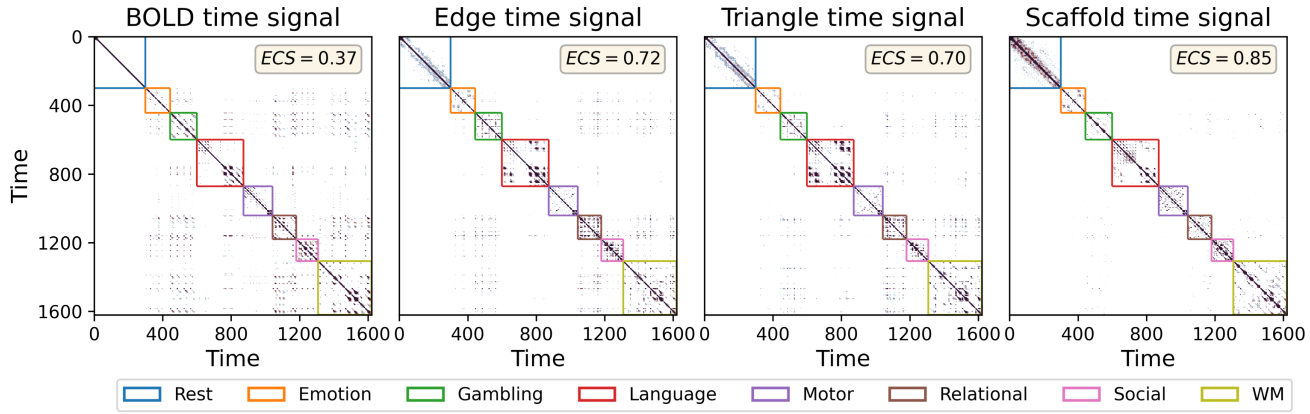


Figure S2: **Local higher-order topological indicators for fMRI task differentiability at** $97th$ **percentile**. As done in the main text, we compute *local* observables by comparing the temporal recurrence plots (i.e. time-time correlation matrices) for the four methods, from the lower-order methods (BOLD and edge signals) to higher-order ones (triangle and scaffold time signals). We set a common threshold at the $97th$ percentile to binarize the data when analyzing an fMRI temporal signal obtained by concatenating resting-state and seven HCP tasks. Colored boxes within the plots denote the ground truth of rest and task segments. The element-centric similarity (ECS) measure provides a quantification of the task decoding ability of the different methods. Here, the scaffold method leads the pack, yet with a lower value than the one reported in the main text. Results are averaged over the 100 subjects, considering the mean between LR and RL phase encoding.

## S2 Brain Fingerprinting

As a second application, we have also considered functional brain fingerprinting [4, 1]. That is, the ability to identify a subject from a group, solely based on their brain's distinctive functional pattern.

We compared the four different methods on the HCP dataset relying on two sessions of resting-state fMRI data (i.e., sessions $REST\_1\_LR$ and $REST\_2\_LR$ for test and retest, respectively) by assessing the quality of fingerprinting using the differential identifiability measure [1]. We report in Fig. S3 the performances of the functional brain fingerprinting across the four methods considered to analyze resting-state fMRI data. In particular, when focusing on whole-brain connections we find similar performances across methods. The performance scores are in line with some previous results [1], but can slightly differ across studies [7] given that identifiability scores are typically influenced by different steps of the fMRI preprocessing, or brain parcellation considered (in this study we considered 19 ROIS among subcortical and cerebellum, for a total of 119 ROIs).

As done in the main text, we can also explore subject-specific patterns of brain activation. We report in Fig. S4 the coefficient of variation (cv) for the triangle approach, when projecting the triangle weights of connections that involve either one (respectively two and three) nodes within one of the seven resting-state functional networks on the cortical brain surface, averaged across the 100 HCP subjects. Interestingly, the interactions between unimodal (i.e. visual, somatosensory) areas and transmodal (e.g. DMN, frontoparietal) brain areas significantly vary between subjects, as indicated by the high values of the coefficient of variation between the seven functional networks.

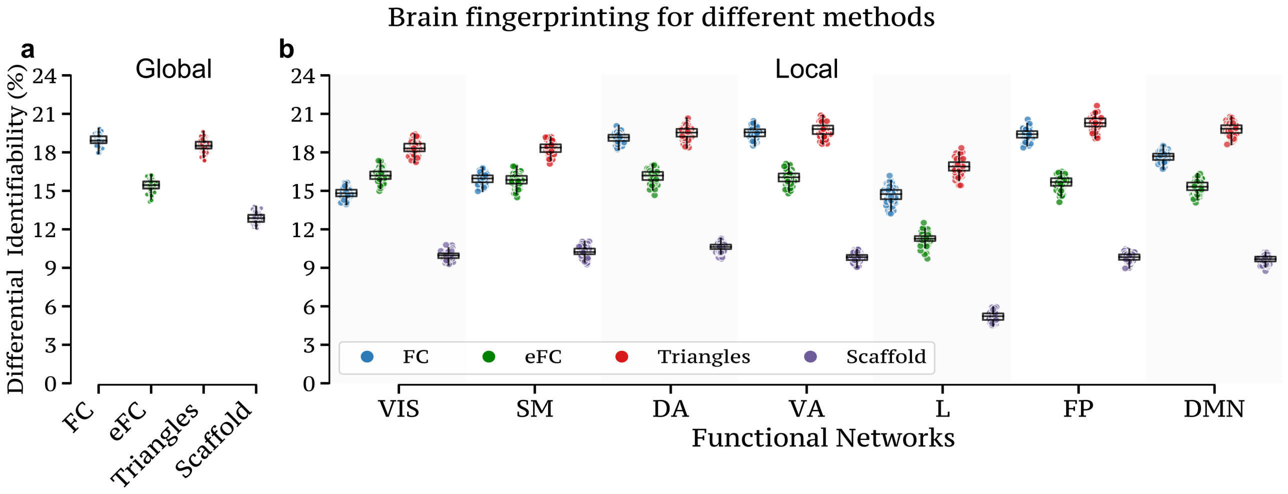


Figure S3: **Global and local functional brain fingerprinting performance across methods**. (**a**) We report the differential identifiability scores obtained when focusing on whole-brain connections. In this scenario, the identifiability scores of the four methods [i.e. Functional Connectivity (FC), edge Functional Connectivity (eFC), triangles, scaffold] exhibit no significant differences, with all methods yielding very similar results, with FC and triangles leading the pack. (**b**) When repeating the analysis considering only specific local connections, we also report the scores obtained when considering the functional connections having at least one node in the functional network analyzed, namely, visual (VIS), somatomotor (SM), dorsal attention (DA), ventral attention (VA), limbic (L), frontoparietal (FP), and default mode network (DMN). Results are obtained by sampling 80 subjects from the total of 100 and repeated this process 100 times ($n = 100$). The box plots show the median and interquartile range (IQR), with whiskers extending to Q1-1.5IQR and Q3+1.5IQR. Individual data points, including outliers, are shown using scatter plots.

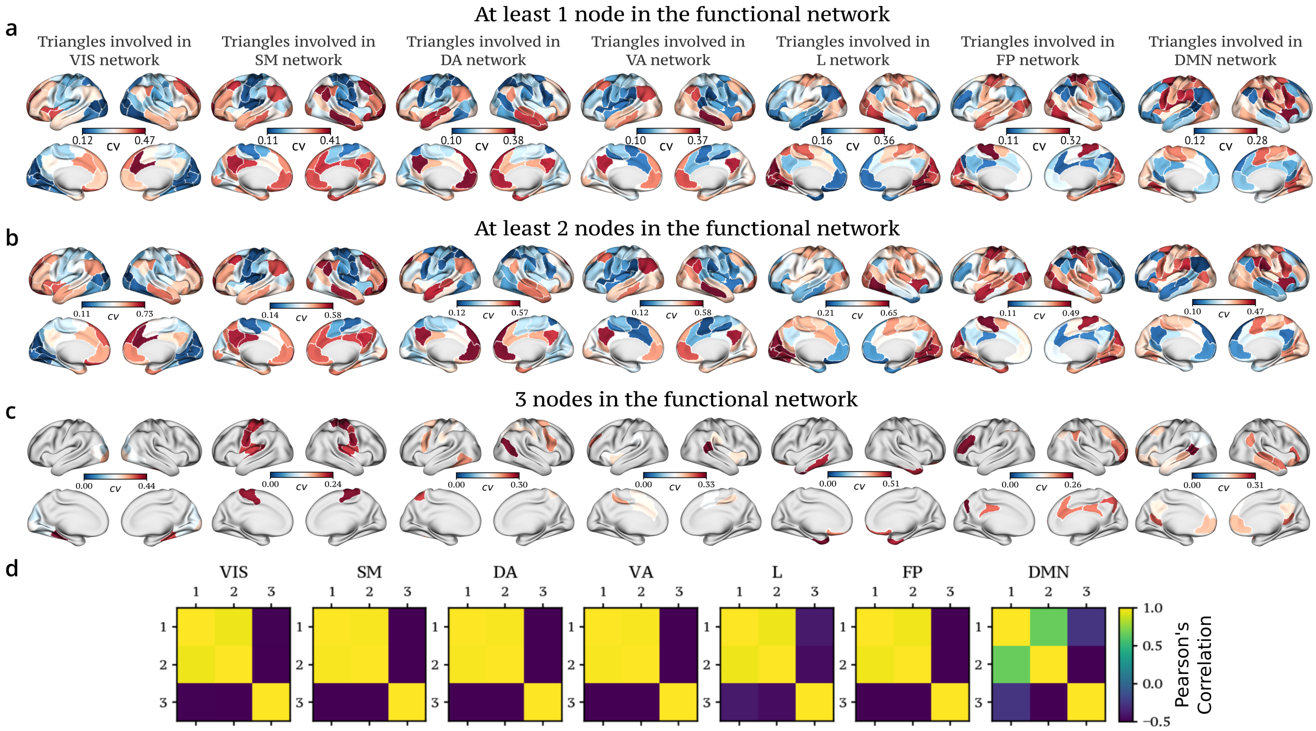


Figure S4: **Cortical brain projections for functional brain fingerprinting**. **(a)** We report the subject-specific brain activation patterns by examining the coefficient of variation (cv) for triangle nodal strength on the cortical brain surface, averaged across the 100 HCP subjects. Specifically, our focus extends to triangles having at least one **(a)**, two **(b)**, or three **(c)** nodes within the functional network of interest. As discussed in the main text, the interaction of somatosensory areas with higher-order networks (i.e., FP and DMN) exhibits considerable variability among subjects, as evidenced by the high values of the coefficient of variation. **(d)** The Pearson's correlation coefficient $\rho$ obtained from comparing cortical maps reveals distinctions only in the way triangle interactions occur within DMN and VIS networks.

## S3 Partial Least Square Correlation (PLSC) and Canonical Correlation Analysis (CCA)

This section delves deeper into the performance of PLSC used in the main text, while also comparing the results against another multivariate method, such as CCA (Canonical Correlation Analysis). We first analyze the total covariance explained by each method using PLSC (same as Figure 4), and the brain-behavior correlation of the significant latent variables.
Figure S5 summarizes the results. Panel **(b)** shows the correlation values between the significant latent components ($p < 0.05$). Here, higher-order approaches occasionally yield slightly higher correlations, suggesting a potential advantage, while displaying a high amount of variance.

Nevertheless, a very recent study raised concerns about the stability of PLSC and CCA when considering a high number of features per subject [6]. To address this, we performed an additional analysis restricting ourselves to a few brain features extracted using the highest Median Absolute Deviation (MAD) value to reduce the number of features, similar to previous work [10]
Figure S6 displays the results of the PLSC analysis for this reduced feature set (i.e. MAD features = 10). While the total covariance values differ from those reported in the main text, the overall trend

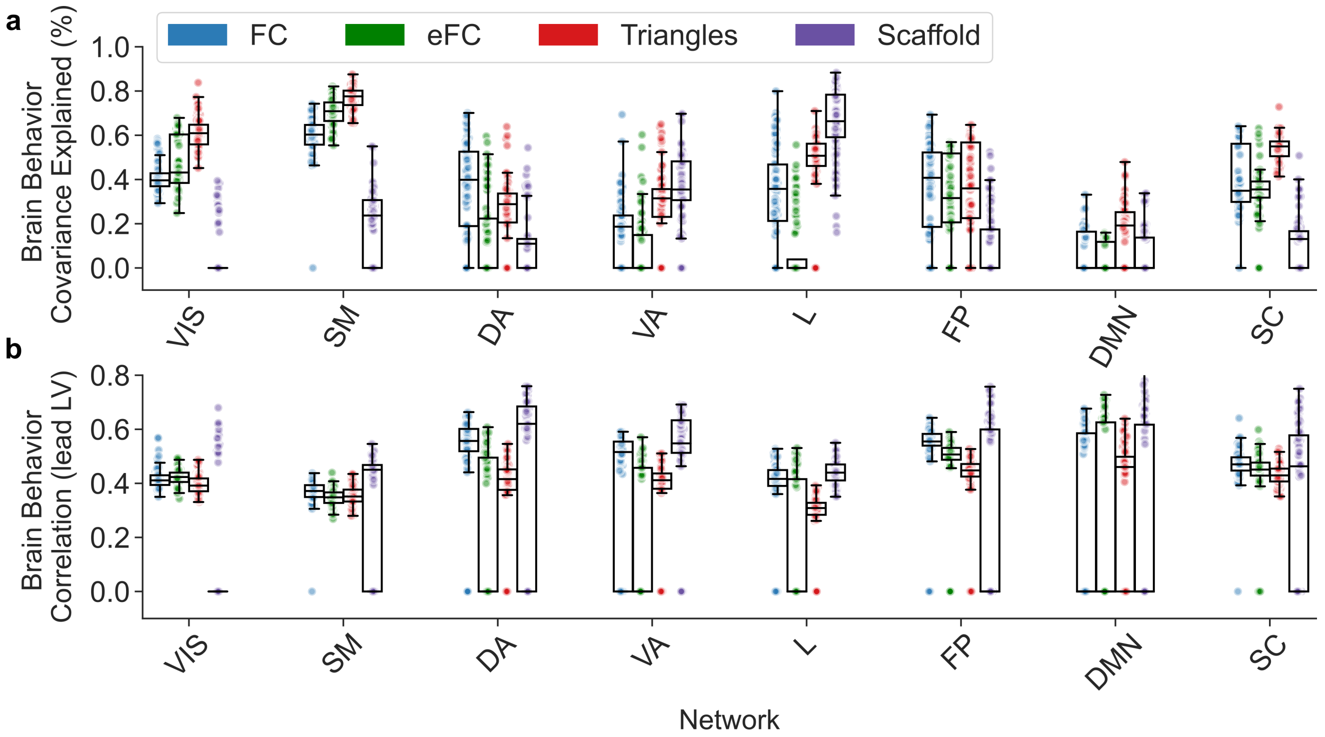


Figure S5: **Behavioral significance across methods for fMRI resting-state data. (a)** Same panel as Figure 4**b**. Percentage of covariance explained by significant latent components ($p < 0.05$) obtained from Partial Least Square Correlation (PLSC) analyses, considering the local functional connections extracted from the different methods and 10 cognitive scores. PLSC components were independently assessed for each method. To obtain a more robust estimate of the covariance explained, we consider a bootstrap procedure randomly sub-sampling 80 subjects from the total of 100 and repeating the PLSC analysis 100 times. **(b)** Correlation between brain and behavioral scores considering the significant latent variables ($p < 0.05$). Higher-order approaches occasionally yield slightly higher correlations, suggesting a potential advantage. Functional networks: visual (VIS), somatomotor (SM), dorsal attention (DA), ventral attention (VA), limbic (L), frontoparietal (FP), default mode network (DMN), and subcortical (SC). The box plots show the median and interquartile range (IQR), with whiskers extending to Q1-1.5IQR and Q3+1.5IQR. Individual data points, including outliers, are shown using scatter plots.

remains consistent. As we move from lower-order methods like functional connectivity (FC) or edge functional connectivity (eFC) to higher-order methods like violating triangles or scaffolds, we observe a significant increase in the explained brain-behavior covariance. The only exception is the FP network. When examining the brain behavior correlation, all methods achieve similar performance, with higher-order approaches providing slightly higher mean correlations in some networks.

We now shift our focus to examining the performance of the four approaches using Canonical Correlation Analysis (CCA) to assess brain-behavior associations. For this comparison, we relied on the R code provided in Ref. [10]. Here, the 100 HCP participants are randomly stratified into a discovery ($n = 67$) and a replication sample ($n = 33$).
It is important to note that PLSC is often favored over CCA when dealing with high-dimensional data, as mentioned in Ref. [6] This preference is due to CCA's sensitivity in situations where one set has significantly more variables ($p$) than the other ($q$), particularly when $p \gg q$. In contrast, PLSC tends to be less affected by this issue and demonstrates greater robustness to noise. While CCA aims to maximize the correlation between latent variables from matrices $X$ and $Y$, PLSC focuses on maximizing covariance,

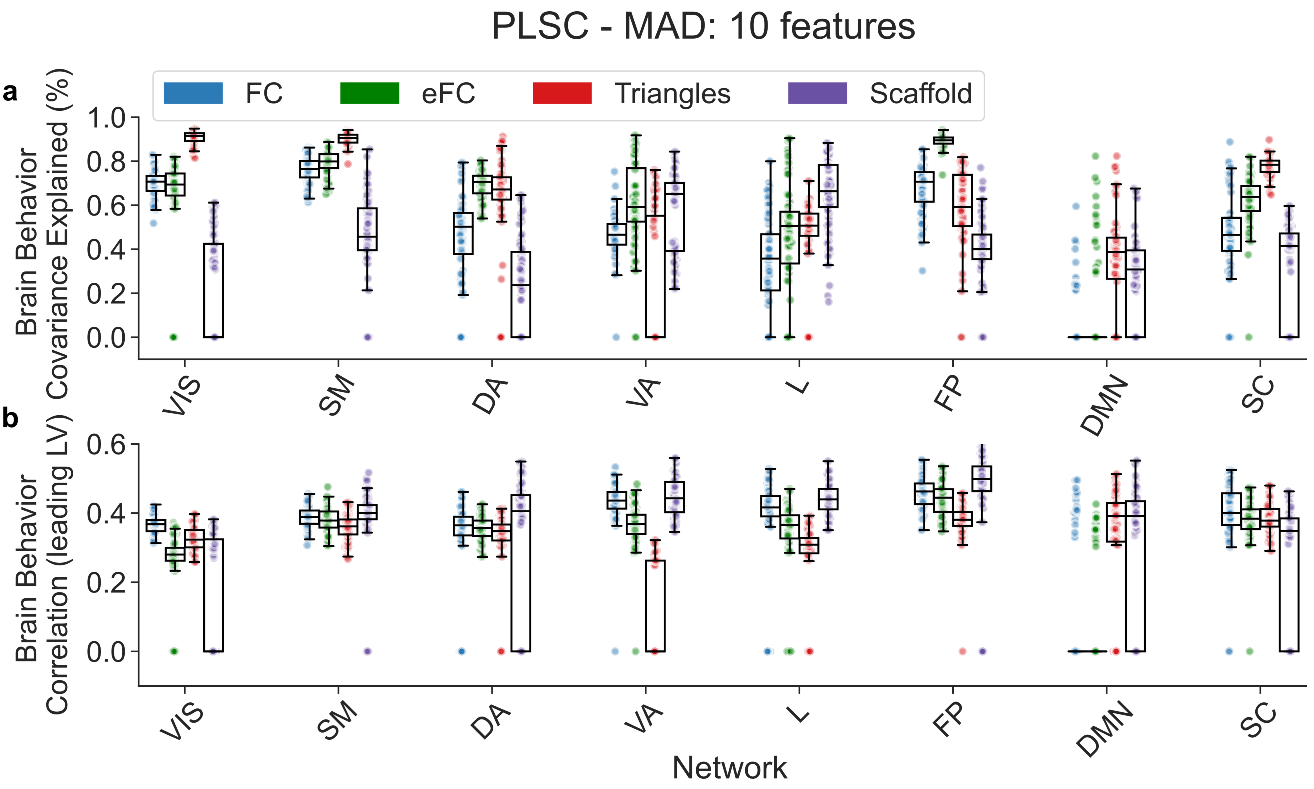


Figure S6: **PLSC behavioral significance across methods considering a reduced number of brain features. (a)** Percentage of the total covariance explained by significant components ($p < 0.05$) obtained from PLSC analyses, considering the top 10 functional connections extracted using Median Absolute Deviation (MAD) and 10 cognitive scores. PLSC components were independently assessed for each method. To obtain a more robust estimate of the covariance explained, we consider a bootstrap procedure randomly sub-sampling 80 subjects from the total of 100 and repeating the PLSC analysis 100 times. **(b)** Correlation score between brain and behavioral scores considering the first significant latent variable ($p < 0.05$). All methods achieve similar performance in this case. Higher-order approaches occasionally yield slightly higher correlations, suggesting a potential advantage. The box plots show the median and interquartile range (IQR), with whiskers extending to Q1-1.5IQR and Q3+1.5IQR. Individual data points, including outliers, are shown using scatter plots.

with X representing brain features and Y representing behavioral features.
Given the potential stability issues of CCA with high-dimensional data [6], we again used the same 10 brain features identified using MAD. Figure S7 summarizes the results of our investigation. In particular, Figure S7**a** reports the canonical correlation induced by the leading significant latent variable. In a similar way as shown for the PLSC covariance analysis, also in this case higher-order approaches typically perform better than lower-order methods, with the only exception of the VA network. Conversely, when focusing on the total covariance explained by the significant latent components ($p < 0.05$), the eFC method performs better in three out of the seven functional resting-state networks (namely DA, VA, and FP), while higher-order approaches lead in the remaining four.

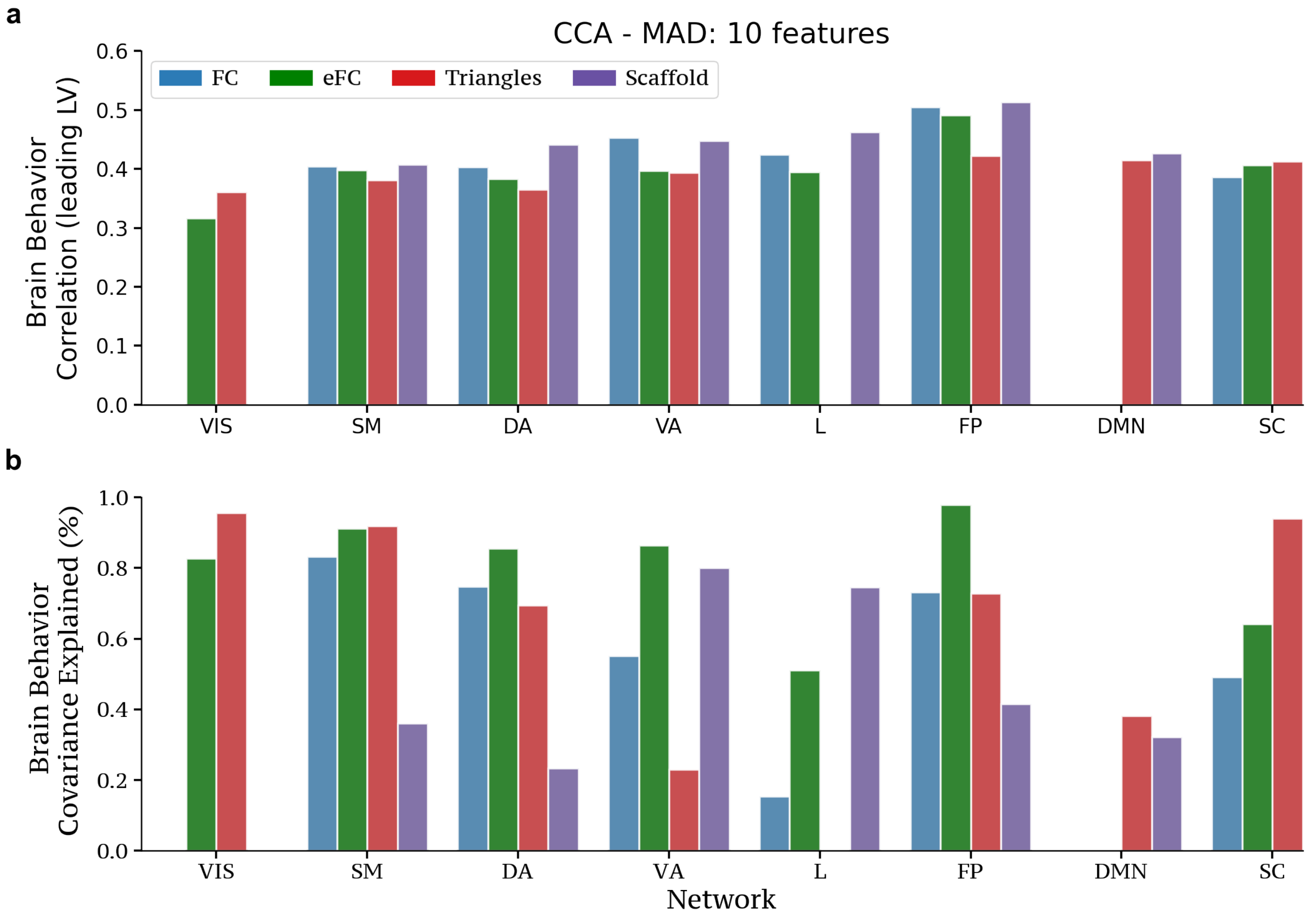


Figure S7: **Behavioral significance across methods using Canonical Correlation Analysis. (a)** Canonical correlation associated with the first significant latent variable ($p < 0.05$, highest covariance explained). For each method, this score was obtained by considering CCA between the top 10 functional connections identified using the MAD metric and 10 cognitive scores. Higher-order methods generally display higher correlation scores, except for VA, suggesting a potential advantage. **(b)** Percentage of covariance explained by the significant latent components ($p < 0.05$) from the CCA analysis. In this case, eFC performed better in three functional networks (DA, VA, and FP), while higher-order approaches excelled in other resting-state functional networks. CCA components were independently assessed for each method. Correlation and covariance estimates were derived by randomly dividing HCP subjects into discovery ($n = 67$) and replication ($n = 33$) sets. Standard errors on the correlation were smaller than 0.01.

## S4 Comparison with multivariate information-theoretic approaches

In this section, we compare the topological approach presented in the main text with recent static higher-order information-theoretic approaches based on O-information, S-information, and Dual Total Correlation (DTC) [8]. We relied on the code provided in Ref. [5] for this comparison.

We evaluated the similarity between the activation patterns of local higher-order measures (violating triangles and the homological scaffold) identified by our topological approach and those identified by the information-theoretic approaches.

We remark a crucial difference between the methods: Our topological approach inherently captures temporal dynamics, therefore we need to average the activation patterns across time for each subject. By contrast, the information-theoretic approaches are computed over the entire window of the fMRI scan (1200 fMRI volumes).

### S4.1 Cortical profiles

Figure S8 shows the results of our comparison. We projected the violating triangles, homological scaffold, O-information, and S-information onto the cortical surface (Schaefer 100), as shown in panels **a-f**. We found a moderate correlation (Pearson's correlation coefficient $\approx 0.42$) between the topological activations and those from the information-theoretic methods. The correlation values vary depending on whether we consider activation at the nodal level (after projection) or at the original triangle level (panels **g** and **h**). Furthermore, brain surface projections of the scaffold-to-triangles ratio and the redundancy-to-synergy gradient scores (based on their respective ranks) also revealed similar cortical patterns ($\rho = 0.41$). Notice that the redundancy and synergy contributions are derived from the O-information vector of size $\binom{N}{3}$ isolating only the positive and negative contributions, respectively. In simpler terms, in resting-state fMRI data, we find that the violating triangle activation pattern moderately resembles redundancy, while the homological scaffold aligns more with synergistic contributions.

### S4.2 Functional brain fingerprinting

This section compares the performance of information-theoretic methods against our topological approach in the context of functional brain fingerprinting. Similar to the analysis in Figure 3 of the main text, we assessed the differential identifiability achieved by each method. Figure S9 shows the results when considering connections involving at least one node within one of the seven resting-state functional networks. Interestingly, the information-theoretic methods outperform the topological methods for all networks except the visual network. This is remarkable, although one needs to consider that the information-theoretic approaches are inherently static, in the sense that for the analysis they require using the entire 1200 fMRI window at once. In contrast, our method obtains activation vectors from instantaneous activation data, which is then averaged across the activation of the individual 1200 volumes (which is also potentially a source of noise or artefacts).

### S4.3 Brain-behavior association

This section compares the performance of information-theoretic methods with our topological approach in uncovering brain-behavior associations, using the PLSC technique. Similar to the analysis in Figure 4 of the main text, we assessed the amount of covariance explained by each method, both globally and locally. Figure S10**a** shows the percentage of total covariance explained by the significant multivariate correlation components when considering all brain connections. In this whole-brain analysis, O-information yielded the highest brain-behavior covariance, with an average value across bootstrapped runs of around 40%. However, when we analyze connections within specific resting-state functional networks (Fig. S10**b**), the results differ. With a few exceptions, such as the dorsal-attention (DA) and frontoparietal (FP) networks, triangles and scaffold measures consistently explained a higher percentage of covariance for brain-behavior associations.

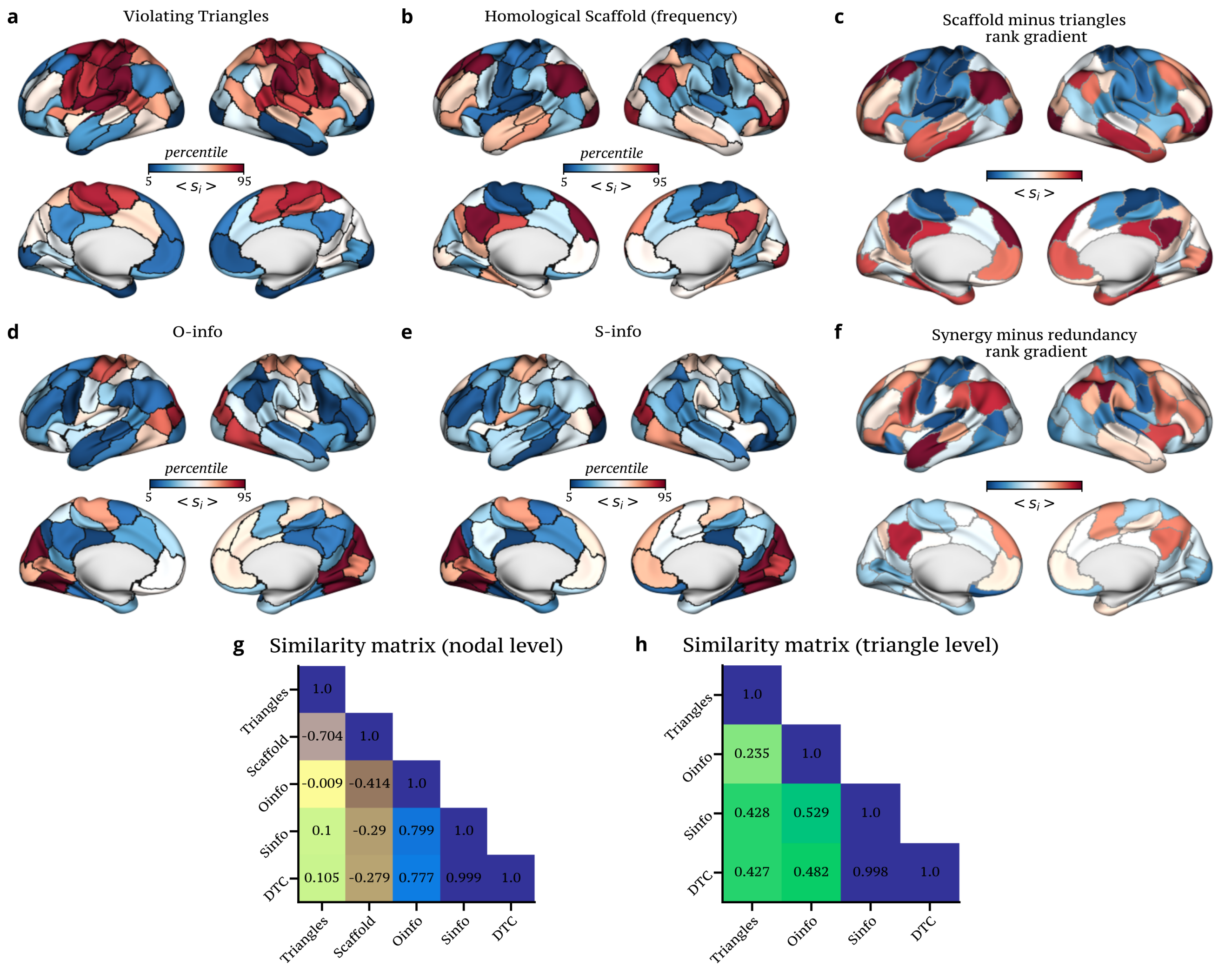


| | Triangles | Scaffold | Oinfo | Sinfo | DTC |
|---|---|---|---|---|---|
| Triangles | 1.0 | | | | |
| Scaffold | -0.704 | 1.0 | | | |
| Oinfo | -0.009 | -0.414 | 1.0 | | |
| Sinfo | 0.1 | -0.29 | 0.799 | 1.0 | |
| DTC | 0.105 | -0.279 | 0.777 | 0.999 | 1.0 |

| | Triangles | Oinfo | Sinfo | DTC |
|---|---|---|---|---|
| Triangles | 1.0 | | | |
| Oinfo | 0.235 | 1.0 | | |
| Sinfo | 0.428 | 0.529 | 1.0 | |
| DTC | 0.427 | 0.482 | 0.998 | 1.0 |

Figure S8: **Cortical profiles of topological and information-theoretic measures**. We report the activation of the local higher-order measures, namely **(a)** violating triangles and **(b)** homological scaffold, and those identified by the information-theoretic approaches, **(d)** O-info and **(e)** S-info, respectively. Brain surface projections of the **(c)** scaffold-to-triangles ratio and the **(f)** redundancy-to-synergy gradient scores (based on their respective ranks) also reveal similar patterns, as confirmed by the Pearson's correlation coefficient of $\rho = 0.42$. We also compare between the topological activations and the corresponding maps obtained from the information-theoretic methods, either at the **(g)** nodal level (after projection) or at the **(h)** original triangle level.

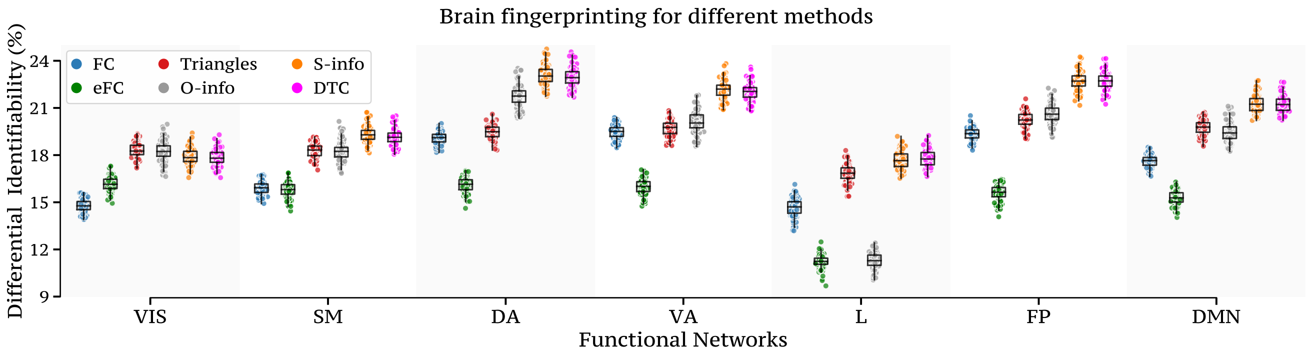


Figure S9: **Functional brain fingerprinting performance across methods for resting-state fMRI data.** For each method, we present the differential identifiability scores for functional connections associated with the following networks: visual (VIS), somatomotor (SM), dorsal attention (DA), ventral attention (VA), limbic (L), frontoparietal (FP), and default mode network (DMN). Except for the visual network, the S-info/DTC metric outperforms all other approaches. The box plots show the median and interquartile range (IQR), with whiskers extending to Q1-1.5IQR and Q3+1.5IQR. Individual data points, including outliers, are shown using scatter plots.

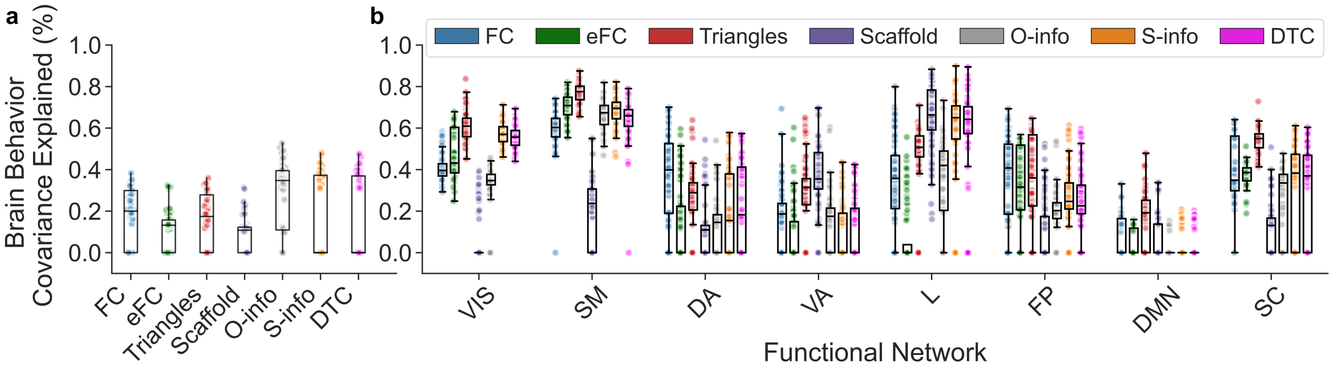


Figure S10: **Behavioral significance across methods for fMRI resting-state data.** **(a)** Percentage of brain cognition covariance explained by significant multivariate correlation components ($p < 0.05$) obtained from PLSC analyses between the whole-brain functional connections extracted from the different methods and 10 cognitive scores. PLSC components were independently assessed for each method. To obtain a more robust estimate of the covariance explained, we consider a bootstrap procedure randomly subsampling 80 subjects from the total of 100 and repeating the PLSC analysis 100 times. On a whole-brain level, O-information performs better than all the other approaches. **(b)** We repeat the same PLSC analysis considering only the functional connections extracted from the different methods within specific functional networks (i.e. all interactions between nodes of the same network). As shown in Figure 4 of the main text, also in this case higher-order methods perform better in terms of covariance explained, reaching 80% in somatosensory areas. Functional networks: visual (VIS), somatomotor (SM), dorsal attention (DA), ventral attention (VA), limbic (L), frontoparietal (FP), default mode network (DMN), and subcortical (SC). Except for the visual network, the S-info/DTC metric outperforms all other approaches. The box plots show the median and interquartile range (IQR), with whiskers extending to Q1-1.5IQR and Q3+1.5IQR. Individual data points, including outliers, are shown using scatter plots.

## S5 ConnICA comparison

This section compares the methods presented in the main text with the ConnICA approach [2, 3]. ConnICA utilizes Independent Component Analysis (ICA) to extract stable independent functional connectivity patterns (FC-traits) from a set of individual functional connectomes, without imposing any *a priori* data stratification into groups. The original ConnICA study investigated the links between FC-traits derived from resting-state fMRI and cognitive/clinical features related to consciousness levels. Here, we apply ConnICA with the goal of identifying stable FC traits that can be used for brain fingerprinting. Similarly to what was done in Ref [2], we set the number of ICA components to 20 and independently applied ConnICA to the resting-state fMRI data of 100 HCP subjects across two sessions (day 1 and day 2). Figure S2 shows the results of ConnICA approach for brain fingerprinting. First, only one FC-trait exhibited a moderate correlation (Pearson's correlation coefficient $rho \approx 0.5$) between the two sessions. All other traits showed low similarity (see panel **a**). We rely on the most robust FC trait between the sessions to quantify whole-brain fingerprinting accuracy. Specifically, we consider Intraclass Correlation Coefficient (ICC) [9] to assess the paired FC-trait weights across subjects (see panel **b**). We remind that ICC is a statistical measure used to determine the agreement between ratings/scores of different groups. In other words, the stronger the agreement between two or more groups, the higher the ICC value. Finally, we reconstruct functional connectomes using only FC-traits with a correlation coefficient above 0.4 for both sessions. This allows us to perform similar analyses to those presented in Figure 3 of the main text. As shown in Figure S2**c**, higher-order approaches outperform ConnICA, which exhibits quite a high variance across multiple bootstrapped runs (sub-sampling only 80 out of 100 HCP subjects at each of the 100 iterations).

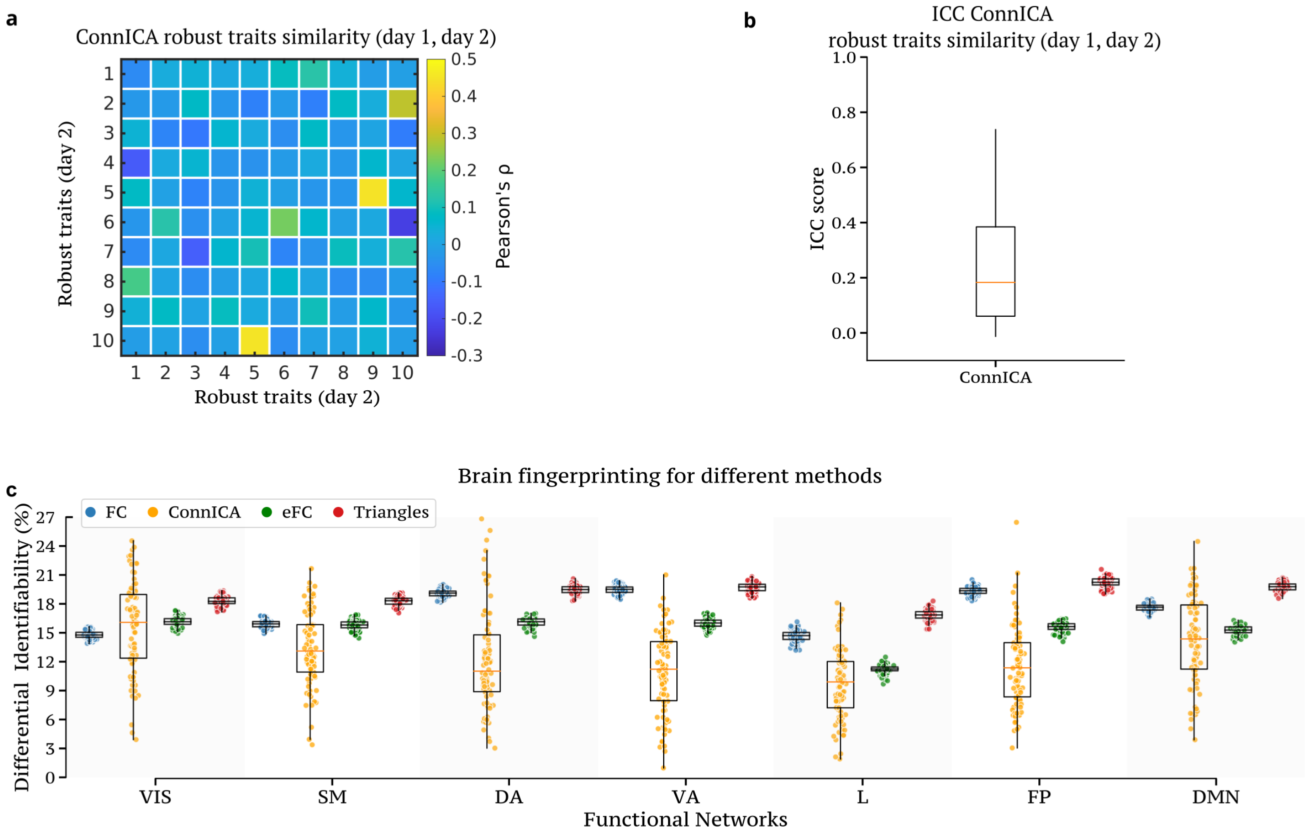


Figure S11: **ConnICA approach for brain fingerprinting**. **(a)** Pairwise Pearson's correlation between all the robust FC-traits independently identified using ConnICA for the two HCP sessions. Only one FC-trait showed a correlation slightly above 0.5, indicating low similarity of the robust FC traits between the sessions. **(b)** Comparing the weights of the robust FC-trait associated with each subject in the two different sessions yields a low Intra-Class Correlation (ICC). This reflects the difficulty of accurately identifying subjects based on FC-traits alone. **(c)** We reconstructed functional connectomes using only FC-traits with a correlation coefficient greater than 0.4 for both sessions. The differential identifiability scores for the ConnICA approach, calculated at the level of the seven resting-state networks, confirm its lower performance compared to the higher-order methods presented in the main text. Additionally, ConnICA exhibits higher variability across multiple bootstrapped runs. Results in panel **b-c** are obtained using only 80 out of the 100 HCP subjects, for 100 independent iterations. The box plots show the median and interquartile range (IQR), with whiskers extending to Q1-1.5IQR and Q3+1.5IQR. Individual data points, including outliers, are shown using scatter plots.

# References

[1] E. Amico and J. Goñi. The quest for identifiability in human functional connectomes. *Scientific Reports*, 8(1):1–14, 2018.

[2] E. Amico and J. Goñi. Mapping hybrid functional-structural connectivity traits in the human connectome. *Network Neuroscience*, 2(3):306–322, 09 2018.

[3] E. Amico, D. Marinazzo, C. Di Perri, L. Heine, J. Annen, C. Martial, M. Dzemidzic, M. Kirsch, V. Bonhomme, S. Laureys, and J. Goñi. Mapping the functional connectome traits of levels of consciousness. *NeuroImage*, 148:201–211, 2017.

[4] E. S. Finn, X. Shen, D. Scheinost, M. D. Rosenberg, J. Huang, M. M. Chun, X. Papademetris, and R. T. Constable. Functional connectome fingerprinting: Identifying individuals using patterns of brain connectivity. *Nature Neuroscience*, 18(11):1664–1671, Nov. 2015.

[5] M. Gatica, R. Cofré, P. A. Mediano, F. E. Rosas, P. Orio, I. Diez, S. P. Swinnen, and J. M. Cortes. High-Order Interdependencies in the Aging Brain. *Brain Connectivity*, 00(00):1–11, 2021.

[6] M. Helmer, S. Warrington, A.-R. Mohammadi-Nejad, J. L. Ji, A. Howell, B. Rosand, A. Anticevic, S. N. Sotiropoulos, and J. D. Murray. On the stability of canonical correlation analysis and partial least squares with application to brain-behavior associations. *Communications Biology*, 7(1):217, Feb 2024.

[7] Y. Jo, J. Faskowitz, F. Z. Esfahlani, O. Sporns, and R. F. Betzel. Subject identification using edge-centric functional connectivity. *NeuroImage*, 238:118204, Sept. 2021.

[8] F. E. Rosas, P. A. Mediano, M. Gastpar, and H. J. Jensen. Quantifying high-order interdependencies via multivariate extensions of the mutual information. *Physical Review E*, 100(3):32305, 2019.

[9] P. E. Shrout and J. L. Fleiss. Intraclass correlations: Uses in assessing rater reliability. *Psychological Bulletin*, 86(2):420–428, Mar. 1979.

[10] C. H. Xia, Z. Ma, R. Ciric, S. Gu, R. F. Betzel, A. N. Kaczkurkin, M. E. Calkins, P. A. Cook, A. García de la Garza, S. N. Vandekar, Z. Cui, T. M. Moore, D. R. Roalf, K. Ruparel, D. H. Wolf, C. Davatzikos, R. C. Gur, R. E. Gur, R. T. Shinohara, D. S. Bassett, and T. D. Satterthwaite. Linked dimensions of psychopathology and connectivity in functional brain networks. *Nature Communications*, 9(1):3003, Aug 2018.
